# Reproducible mini-slump test procedure for measuring the yield stress of cementitious pastes

**DOI:** 10.1617/s11527-017-1103-x

**Published:** 2017-10-19

**Authors:** Zhijun Tan, Susan A. Bernal, John L. Provis

**Affiliations:** Department of Materials Science and Engineering, The University of Sheffield, Sir Robert Hadfield Building, Mappin St, Sheffield, S1 3JD UK

**Keywords:** Mini-slump test, Yield stress, Workability, Viscometry, Alkali-activated cements

## Abstract

The mini-slump test is a fast, inexpensive and widely adopted method for evaluating the workability of fresh cementitious pastes. However, this method lacks a standardised procedure for its experimental implementation, which is crucial to guarantee reproducibility and reliability of the test results. This study investigates and proposes a guideline procedure for mini-slump testing, focusing on the influence of key experimental (mixing and testing) parameters on the statistical performance of the results. The importance of preparation of always testing at the same time after mixing, testing each batch once rather than conducting multiple tests on a single batch of material, is highlighted. A set of alkali-activated fly ash-slag pastes, spanning from 1 to 75 Pa yield stresses, were used to validate the test method, by comparison of calculated yield stresses with the results obtained using a conventional vane viscometer. The proposed experimental procedure for mini-slump testing produces highly reproducible results, and the yield stress calculated from mini-slump values correlate very well with those measured by viscometer, in the case of fresh paste of pure shear flow. Mini-slump testing is a reliable method that can be utilised for the assessment of workability of cements.

## Introduction

For a given cementitious material in the fresh state, the yield stress (denoted *τ*_*y*_) and the plastic viscosity are generally considered the two most important rheological properties in terms of workability. The yield stress of a cementitious material denotes the critical stress value at which the material will begin to, or cease to, flow, which is an important property when placing the material. Concrete with high yield stress is difficult to pump, and the associated poor workability results in quality control issues in the hardened material [1]. Therefore, the determination of the yield stress of cementitious materials is of great importance in enabling the design of mix formulations with desired workability.

Rheometers are increasingly adopted to determine the yield stress of cement paste or concrete, and there are two methods commonly applied using such instruments to measure yield stress [2]. The first is by ramping the shear rate generated by the rheometer and recording the resultant shear stress, or vice versa. Using rheological models such as the Bingham, Herschel-Bulkley or Casson models [3], the yield stress can be extrapolated from the relationship between the shear rate and shear stress; where the downwards ramp is used in the determination, as is the case here, the quantity measured is the dynamic yield stress. Alternatively, in a ‘direct measurement’ of yield stress, a constant low shear rate (e.g. 0.1–0.001 s^−1^) is applied to the material starting from rest, and the (static) yield stress is identified as the maximum in the stress-time profile [4, 5]. Other methods such as creep recovery can also be used to measure yield stress characteristics [6].

However, measuring yield stress using a rheometer is sometimes difficult due to challenges in the inherent nature of the yield stress material and the proper selection of a rheological model. Problems such as slip flow [7], fracture, and expulsion of the material are commonly encountered when using conventional rheometers in the low shear rate range [3]. Extrapolating yield stress from rheological models relies on the validity of the model, which would be a challenge for a material without an appropriately validated model to describe its rheological behaviour, as is usually the case for non-Portland cements. In addition, yield stress values lower than 10 Pa directly measured by conventional viscometers are commonly less reliable [8] due to the limited sensitivity of most instruments. This range is typical of self-compacting concretes and pastes. Furthermore, from a practical viewpoint, a rheometer is neither inexpensive nor particularly convenient for on-site measurement by field personnel. Conversely, the traditional slump test is still an easy and simple method to study the workability of concretes. It has long been widely used on construction sites because of its simplicity, applicability and very low cost, in spite of the known limitations of repeatability and precision associated with the standard forms of this test [9–11].

In a typical slump test, a mould of a given conical shape is filled with the material to be tested [12]; various different cone geometries and sizes are used in different applications, but in the field of construction materials science the most common is the “Abrams Cone” geometry of ASTM C143 [13], a truncated cone with height:base diameter:top diameter ratios of 3:2:1. Neglecting any inertial effects [14] (when inertia stress is typically ~ 3 Pa), the material flows after the mould is lifted, and then stops when the shear stress due to flow under gravity decreases to the value of the yield stress of the flowing material. Two geometrical quantities are commonly measured: the “slump” and/or the “spread”. The former is the difference between the height of the mould at the beginning of the test and the remaining pat of material when the flow stops (and is a more useful measure for less-fluid pastes), while the latter is the final diameter of the pat of material when flow stops (or after a specified time), and is used to characterise more fluid pastes. The result of the slump test result is at least capable of qualitatively predicting the workability of concrete flow for the purpose of quality control.

However, it is often far from convenient to produce and manipulate the large quantities of material required for a full-scale concrete slump test in a laboratory context, as much research work is conducted with paste or mortar specimens. In order to resolve this issue, the so-called mini-slump test, which is essentially a down-scaled slump test [15], is here examined in detail as a method by which the workability of cementitious pastes may be investigated.

The mini-slump method is a simple, inexpensive and fast test to study the rheology of cement paste, if it can be shown to be applicable and reproducible. Mini-slump test results have previously been quantitatively linked with yield stress values obtained from theory and numerical modelling [16–19], showing the potential for the mini-slump test method to provide valuable information regarding the yield stress of cementitious pastes. However, there is not yet a standard protocol describing the appropriate procedure for performing mini-slump tests on cementitious materials. Inappropriate practical operation of this method could lead to either less reliable or very scattered mini-slump test results, making it difficult to estimate reliable values of the yield stress of the paste. To fill this gap, this paper aims to present a practical procedure for performing mini-slump testing. The study also contributes to the evaluation of workability of cementitious paste especially with very low yield stress, which may be outside the normal measurement range of a conventional viscometer. This is of significant importance in the case of non-Portland cement pastes, such as alkali activated cement paste, as their workability can be very sensitive to factors such as mix formulation (e.g. type and concentration of activator), reaction kinetics, time-dependent behaviour of fresh paste [20] and mixing procedure [21], and the correlation between flow table and rheological test results for these materials has been shown in the past to be problematic [22]. So, a series of alkali-activated pastes are used here as a test case for the proposed protocols.

## Experimental programme

### Materials characterisation and mix designs

**Fig. 1:**
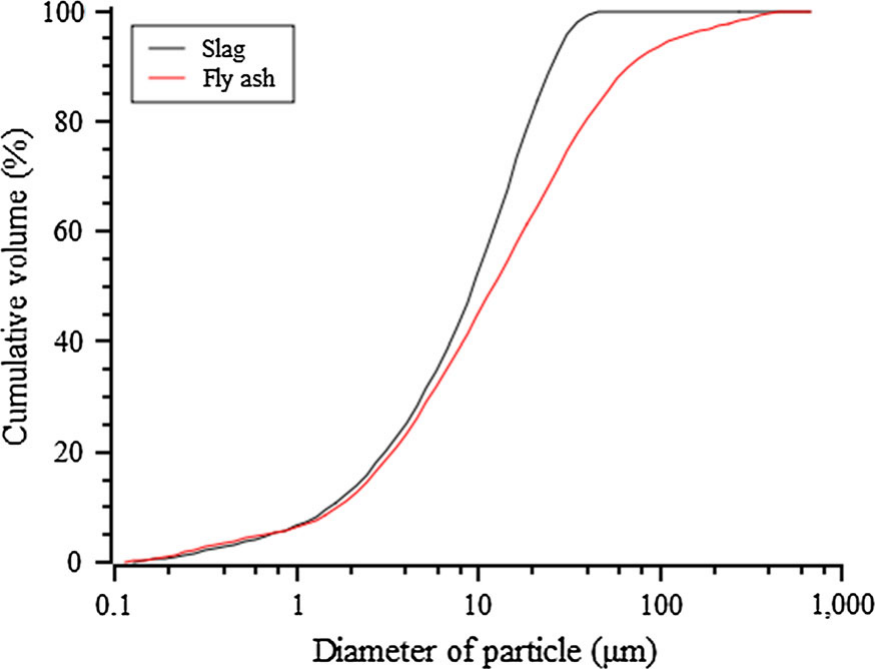
Particle size distribution of slag and fly ash as determined by laser diffractometry, The *D*_*v*_(90) values of the slag and fly ash are 28.6 and 80.1 μm, respectively

Ground granulated blast-furnace slag (ECOCEM, France) and fly ash (BauMineral GmbH, Germany) were used as precursors to prepare the alkali activated cement pastes. Table 1 lists the chemical compositions of the two materials used as determined by X-ray fluorescence (XRF). Figure 1 shows the particle size distributions of the two materials characterised by laser diffraction (Malvern Mastersizer 2000).

**Table 1:** Chemical compositions of the slag and fly ash on an oxide basis, as determined by XRF

Oxide	Slag (wt%)	Fly ash (wt%)
CaO	43.0	5.8
Al_2_ O_3_	10.1	22.7
SiO_2_	37.5	54.5
MgO	6.50	2.5
Na_2_O	0.1	0.9
K_2_O	0.3	1.8
Fe_2_O_3_	0.4	7.3
TiO_2_	0.5	0.9
Mn_3_O_4_	0.2	< 0.05

A total of 27 paste formulations were studied. The total water to binder (where ‘binder’ is defined as precursor + solid component of the activator) ratios were 0.40, 0.44 and 0.48, with activator doses of 4, 8 and 12% relative to the mass of precursor (dry solids basis). As solid precursors, slag and fly ash were blended at different levels (100:0, 75:25 and 50:50 ratios of slag:fly ash by mass) to yield pastes with diverse rheological properties. The alkaline activator was based on a commercial sodium silicate solution (PQ Silicates, UK), composed of 14.7 wt% Na_2_O, 29.4 wt% SiO_2_ and 55.9 wt% H_2_O; the modulus of the activating solution (Ms = SiO_2_/Na_2_O molar ratio) was adjusted by mixing the commercial solution with analytical grade solid NaOH (Sigma Aldrich, UK) to obtain a sodium metasilicate (Ms = 1) solution. The adjusted metasilicate solution was stirred for 2 h, and allowed to cool to ambient temperature, after addition of the NaOH pellets. The solution was used on the same day as prepared, to avoid precipitation of solid sodium metasilicate hydrates. The complete 27 formulations are summarised in Table 2.

**Table 2:** The 27 paste formulations studied; each cell contains the activator doses (in wt% relative to the mass of precursor) used for the specified *w*/*b* and slag:FA ratios

Slag:FA	*w*/*b*		
	0.40	0.44	0.48
100:0	4, 8, 12%	4, 8, 12%	4, 8, 12%
75:25	4, 8, 12%	4, 8, 12%	4, 8, 12%
50:50	4, 8, 12%	4, 8, 12%	4, 8, 12%

### Mixing procedure and testing methodology

**Fig. 2:**
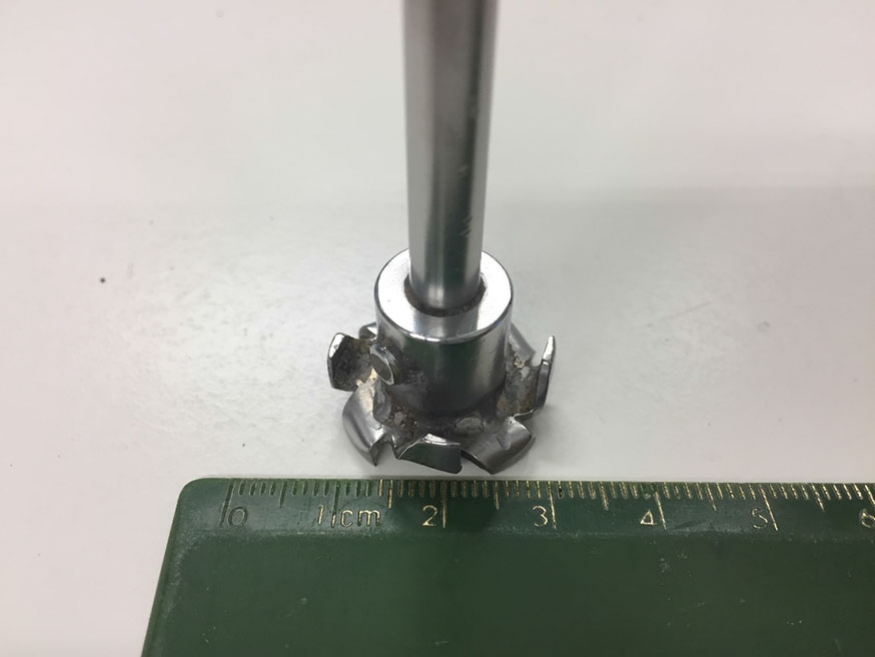
The high shear mixer blade used in this study

For blended pastes containing both slag and fly ash, 30 min of pre-mixing of the dry powders was performed prior to mixing with the activating solution. For each mini-slump test, 60 g of precursor powder was combined with the specified amount of distilled water and activator in a plastic cylindrical container (diameter 5 cm and height 10 cm). The paste was then mixed using a high shear mixer as shown in Fig. 2, at 400 rpm and ambient temperature (20 °C) for 2 min, unless specified otherwise in the description of the influence of testing parameters in Sect. 3 below. The homogeneously mixed paste was immediately used for mini-slump testing, unless specified otherwise in Sect. 3.

**Fig. 3:**
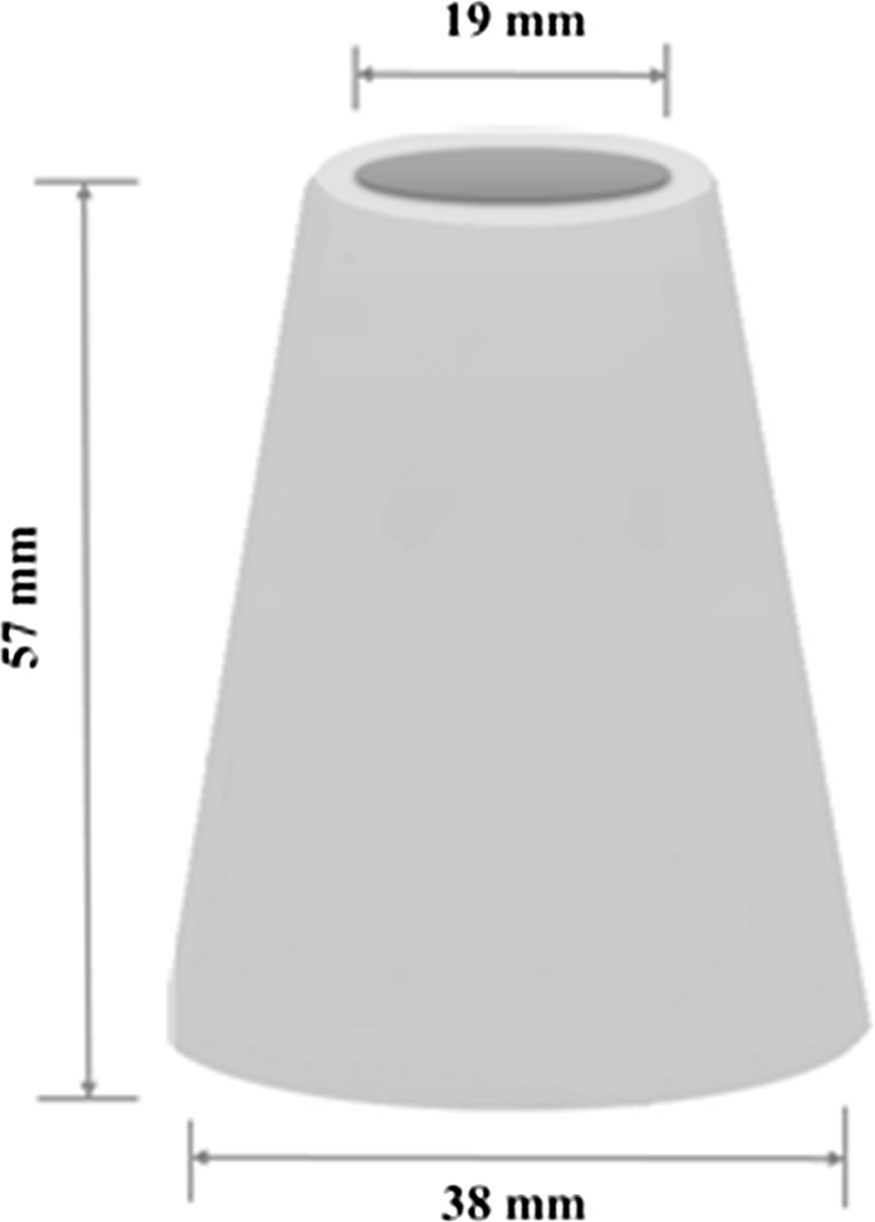
Schematic diagram of mini-slump test cone geometry. Note that the top and bottom diameters are inner sizes of the cone

The mini-slump test setup used in this study is a downscaled Abrams cone geometry [15]: 19 mm top diameter, 38 mm bottom diameter, 57 mm height as shown in Fig. 3. Each test was performed using a poly(tetrafluoroethene) mini-slump cone on a flat sheet of poly(methyl methacrylate) marked with grid squares of 2 × 2 cm^2^. Freshly mixed paste was immediately poured into the cone, and then the cone was lifted as slowly as possible (< 0.005 m/s [23]) to minimise inertial effects. After 1 min, a digital photograph was taken from directly above the resultant pat of material, from which the slump area was calculated using the ImageJ software, calibrated using the scaled grid. The diameter of the spread was then calculated from the slump area. Each paste was tested in triplicate, using three separately mixed batches of paste to obtain good statistical reproducibility of the data obtained, and to ensure that all tests were conducted on pastes of directly comparable shear history.

**Fig. 4:**
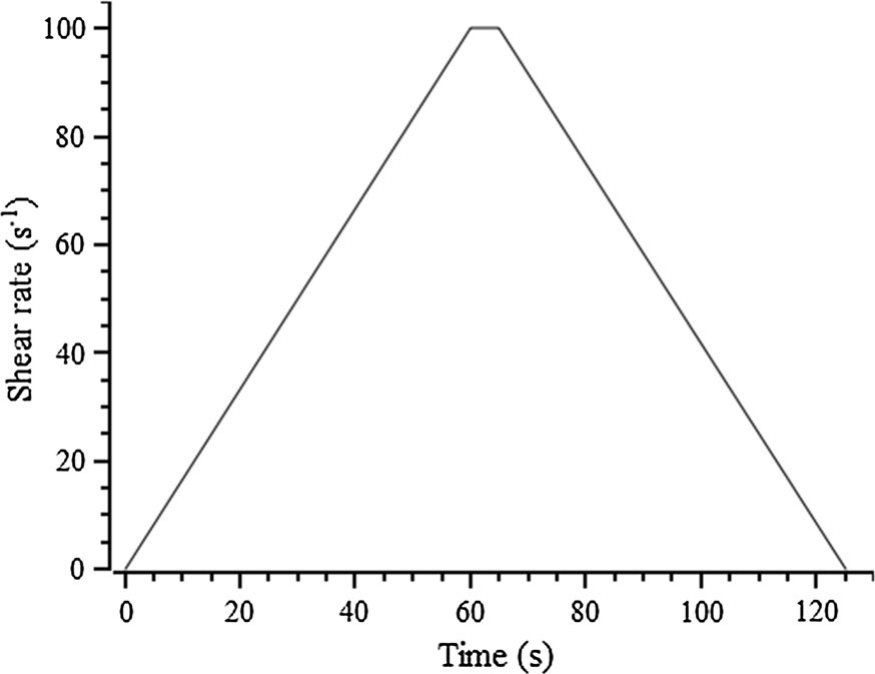
The applied shear rate as a function of time during the viscometric testing

To compare the yield stress values calculated from mini-slump testing with that determined using a viscometer, a HAAKE Viscotester 550 instrument was employed to directly measure the yield stress of fresh pastes, using a six-blade vane-in-cup geometry. During the test, the shear rate was linearly ramped up from 0 to 100 s^−1^ over 60 s, then held for 5 s, then linearly ramped down from 100 s^−1^ to 0 over another 60 s, as illustrated in Fig. 4. The decreasing shear rate-shear stress profile was chosen for analysis, and the yield stress value was obtained by extrapolating the shear rate-shear stress curve to zero shear stress using the Herschel–Bulkley model (Eq. 1), ensuring non-negativity of all parameters during fitting, to obtain the (dynamic) yield stress *τ*_0_.

1 $$\tau = \tau_{0} + k\dot{\gamma }^{n}$$

In Eq. 1, *k* and *n* are fitting constants determined by regression of the shear rate-shear stress curve; the Bingham model is a special case of the Herschel–Bulkley model with *n* = 1.

This method was found to be more reliable and reproducible than the direct measurement of yield stress using a constant low shear rate, particularly for pastes of low yield stress, considering the sensitivity and capabilities of the viscometer used here.

Due to the somewhat larger volume of paste required for the viscometer to achieve pseudo-infinite medium conditions, the pastes to be tested by this method needed to be prepared in a slightly different manner from those used in the mini-slump testing. For each batch to be tested using the viscometer, 270 g of precursor powder was combined with the required amount of distilled water and activator in a 500 mL plastic beaker. The paste was then mixed using the high shear mixer (Fig. 2) at 800 rpm for 2 min, and immediately analysed. The selection of the rotational speed of 800 rpm for the larger batch (270 g precursor cf. 60 g for mini-slump testing) is based on the observation that this mixing protocol was found in preliminary testing to result in equivalent performance (comparable spread sizes of mini-slump test) compared with mixing the 60 g batch at 400 rpm for the same time period (2 min). This indicates that each unit of paste received comparable shear energy from the two mixing strategies.

## Results and discussion

### The influences of mix design parameters on the workability of alkali-activated pastes

Table 3 presents the spread diameters of the 27 alkali-activated pastes measured by mini-slump testing, which range from 72.5 to 139.5 mm across the pastes assessed. None of the pastes showed noticeable bleeding or segregation during the mini-slump test. Increasing the water to binder ratio raised the spread diameters of all pastes, as expected. Under otherwise comparable conditions, i.e. the same water/binder ratio and same slag/fly ash ratio, a higher activator dose also increased the spread diameter due to the dispersion effect of the sodium metasilicate added as activator. Sodium silicate is known to act as a dispersant in particulate suspensions [24, 25], lowering the yield stress of the paste [26] which resulted in a greater spread in the mini-slump test. This dispersion effect is particularly remarkable in the relatively sticky slag-based pastes; the spread diameter is only 72.5 mm in the pure slag paste with 4 wt% activator dose at *w*/*b* 0.40, while a 12 wt% activator dose in the equivalent paste increased the spread diameter to 120.9 mm.

**Table 3:** The mini-slump spread diameters (reported in mm) of alkali-activated slag/fly ash blended pastes

Content of slag in the binder (wt%)	Concentration of activator (wt%)	Water/binder ratio		
		0.40	0.44	0.48
100	4	72.5 ± 1.2	92.7 ± 2.0	105.6 ± 0.5
	8	105.0 ± 0.4	113.7 ± 1.2	118.4 ± 0.7
	12	120.9 ± 1.6	124.8 ± 1.3	134.3 ± 2.4
75	4	95.7 ± 2.3	109.4 ± 1.2	117.7 ± 0.8
	8	109.4 ± 0.9	119.1 ± 0.6	125.7 ± 0.4
	12	135.0 ± 1.7	135.9 ± 1.6	136.2 ± 0.7
50	4	107.2 ± 1.1	117.2 ± 0.5	121.9 ± 0.2
	8	117.9 ± 1.0	124.3 ± 0.5	128.1 ± 0.7
	12	139.5 ± 1.6	137.8 ± 2.9	137.6 ± 0.6

Error values correspond to one standard deviation of three independent measurements

### The correlation of yield stress calculated from mini-slump spread size and measured using a viscometer

Based on the work of Kokado et al. [17] and of Roussel and Coussot [16], the yield stress of pastes can be calculated from the mini-slump spread diameter (or radius) when a set of conditions are fulfilled [14]:

1. The tested volume must be representative of the mixture.
2. The thickness of the sample must be at least five times the size of the largest particle.
3. The surface tension and inertial effects must be negligible.

With the exception of the paste made with 100% slag, at *w*/*b* 0.40 and 4% activator dose, the alkali-activated paste formulations studied here appeared to behave as pure shear flow pastes (i.e. the thickness of spread is far less than the diameter of the spread) [16]. So, the yield stress (*τ*_0_) of each paste was calculated according to Eq. 2 [14, 16, 17], describing the yield stress as a function of the density of the paste *ρ*, volume of the mini-slump cone Ω, and the mini-slump spread diameter *R*.

2 $$\tau_{0} = \frac{{225\rho g\varOmega^{2} }}{{128\pi^{2} R^{5} }}$$

The density of each fresh paste was calculated based on the mix formulation and density of each component, i.e. slag, fly ash, activator solution and water; *g* is the acceleration due to gravity (9.81 m/s^2^).

**Fig. 5:**
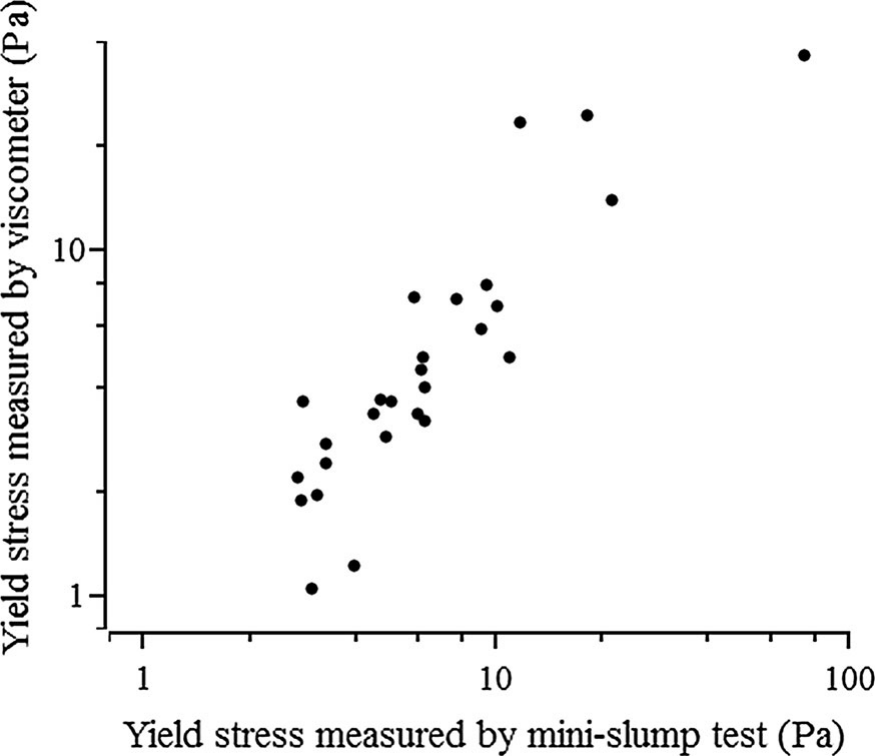
Yield stress values measured by mini-slump test and viscometer

To determine the accuracy of the yield stress values calculated from mini-slump testing of these alkali-activated pastes, the yield stress was also measured using a viscometer, as described in Sect. 2.2. Figure 5 shows the yield stress values obtained according to the two methods. The yield stresses of all pastes range from 1.05 to 74.6 Pa, decreasing as the fly ash content, water to binder ratio and activator dose increase, consistent with the discussion above. Comparison of the results in Fig. 5 shows that low yield stresses (below 10 Pa) determined from the mini-slump test generally correlate very well with viscometer measurements, while there is quite a degree of scatter in the values for yield stresses above 10 Pa, which will be discussed in more detail below. The good agreement between the two methods for low yield stress values demonstrates the applicability and reliability of the mini-slump test for measuring the yield stress of pastes which slump in pure shear flow (i.e. which comply with the conditions noted above for Eq. 2 to be valid), when using the proposed mixing procedure.

However, it is worth noting that yield stress values measured via the mini-slump test are systematically slightly higher than those obtained from the viscometer. This could be attributed to the fact that the effect of the surface tension of the paste was not included in Eq. 2; this would normally be of the same order as the yield stress (1–2 Pa) for low concentration suspensions [27]. The neglect of surface tension effects in the calculation of yield stress using Eq. 2 could systematically overestimate the results compared with those measured by the viscometer. However, Roussel et al. [27] reported that the surface tension effect in the mini-slump test is negligible in pastes with yield stresses larger than about 1 Pa, which is the case for all pastes studied here.

**Fig. 6:**
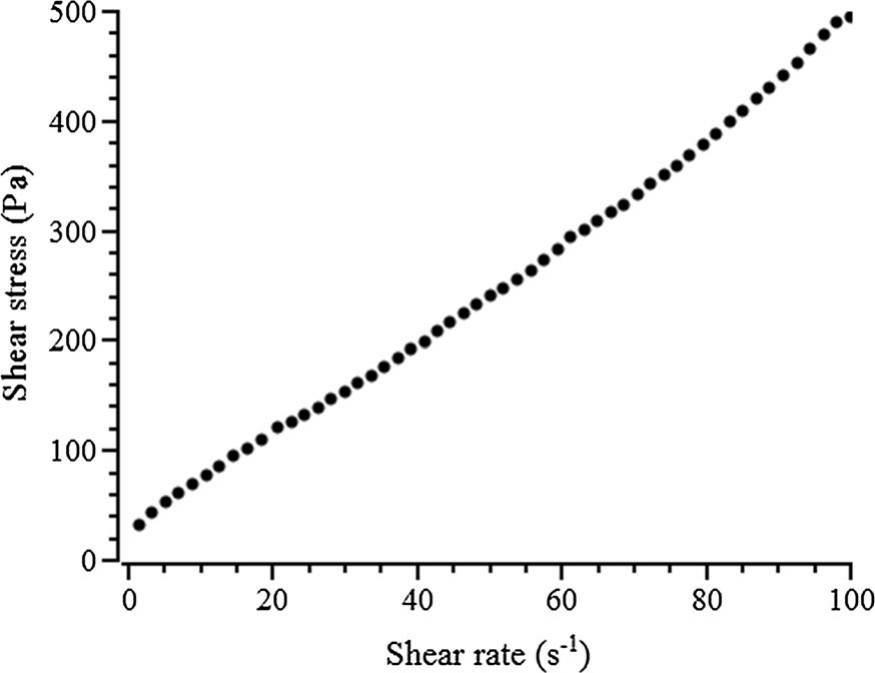
The shear stress-shear rate profile of a paste with 100% slag, *w*/*b* = 0.40 and activator dose 4%, measured by viscometry in a descending ramp (the shear rate linearly decreased from 100 s^−1^ to 0 during 60 s), showing Bingham-type paste behaviour and a yield stress of 36.4 Pa

**Fig. 7:**
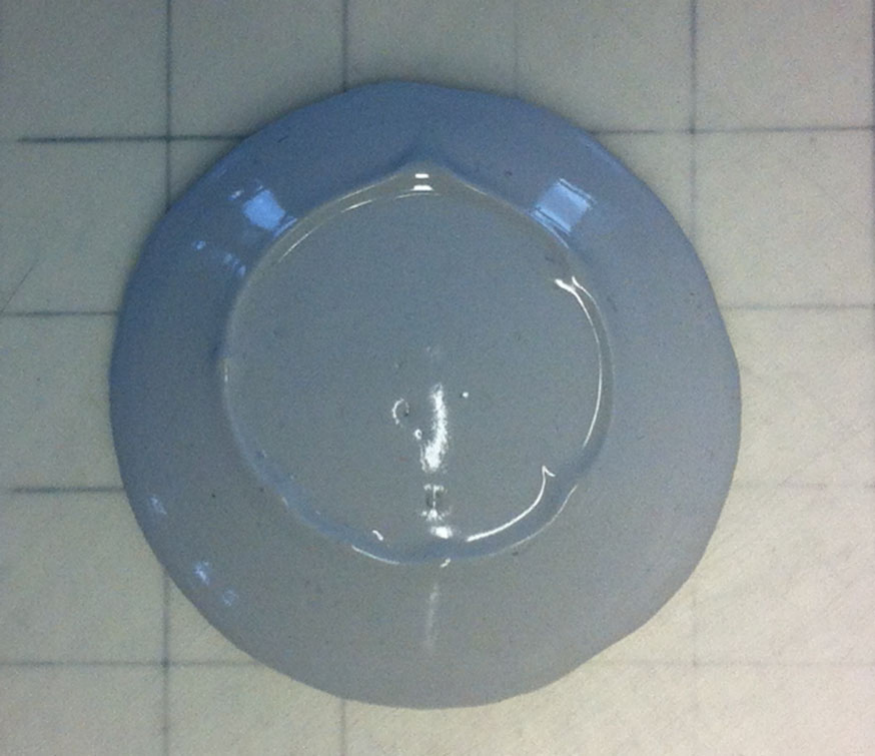
The shape of the pat of a paste with 100% slag, *w*/*b* = 0.40 and activator dose 4% after mini-slump testing, showing evident deviation from uniform shear flow via the raised ring of material in the position of the original cone

At higher yield stress values (> 10 Pa for the pastes in this study), the determination of yield stress by mini-slump testing via Eq. 2 seems less reliable. For example, the yield stress of paste made of 100% slag, *w*/*b* 0.40 and 4% activator dose is 74.6 Pa, which is much higher than that determined by viscometry for the same paste, 36.4 Pa. The latter value is expected to be more accurate and reliable as the viscometer performs very well in this range, and the shear rate-shear stress curve measured by the viscometer (Fig. 6) shows that the rheological model of the paste is well approximated by the Bingham model, giving a relatively reliable determination of the yield stress. The most likely reason for the inaccuracy of the evaluation of yield stress based on mini-slump testing for this paste is that Eq. 2 is derived for pastes undergoing pure shear flow, while the shape of the pat of the paste (Fig. 7) indicates that the paste has undergone neither pure shear flow nor pure elongational flow, which agrees with the conclusion of Pierre et al. [18] that a material is mainly in a spread regime below yield stress values of 18 Pa. Figure 7 shows a visible raised ring of material which has retained the circular form of the cone at the centre of the pat as the material has slumped, which is not consistent with the uniform pat shape resulting from pure shear flow.

### Reproducibility of the proposed procedure for mini-slump testing

**Fig. 8:**
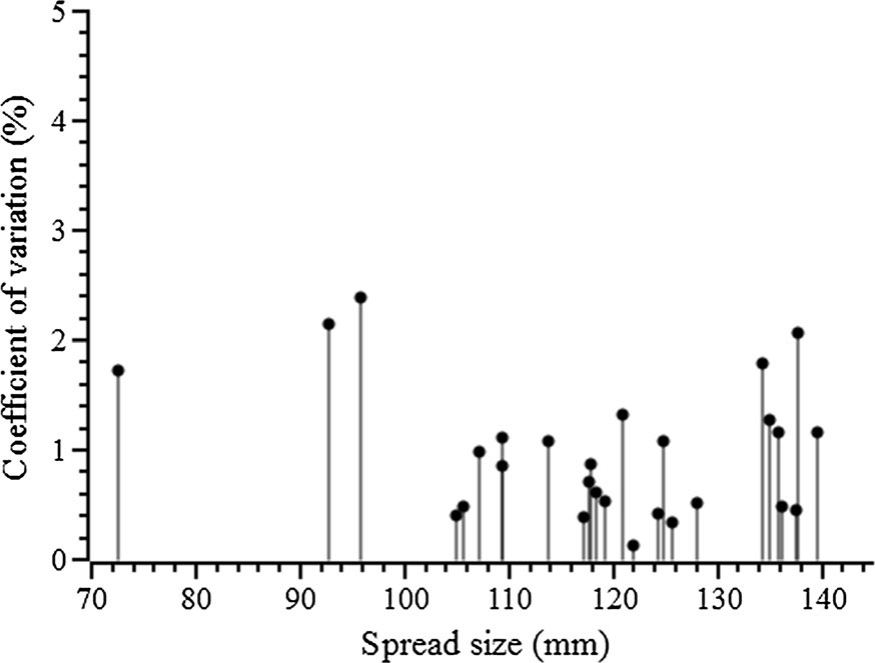
The coefficients of variation (relative standard deviation) of the spread size of all samples

Figure 8 shows the coefficients of variation of spread diameter in three replicates of each of the 27 pastes in the mini-slump test; all values are lower than 2.4%, with 24 of the 27 values lower than 2.0%. The average coefficient of variation of all samples is less than 1%. This demonstrates remarkably good repeatability of the mini-slump testing procedure proposed in this study.

The reproducible measurement of paste spread size in the mini-slump test can also lead to relatively low scatter in the yield stress calculated by Eq. 2. Because this equation involves an inverse 5th-power relationship between yield stress and spread diameter, a 2% coefficient of variation in the spread size can lead to the calculated yield stress varying by approximately 10%, or about 5% when the coefficient of variation in spread is 1%. Considering the low yield stresses associated with pure shear flow of a paste, which is the regime in which Eq. 2 is valid, even a 10% uncertainty in the calculated yield stress would still be a valuable result in terms of a rapid and inexpensive test for the determination of the shear stress of cementitious pastes. However, the ability to achieve such a high reproducibility is contingent on correct use of the test methodology, and so the influence of various test parameters on the ability to obtain correct results will be explored in more detail in the following sections.

### Influence of testing procedure on the results of mini-slump test

Although many aspects of the experimental protocol may affect the statistical performance of mini-slump test results, Pashias et al. [28] reported that no measurable difference was observed in their tests on mini-slump of red mud due to factors such as the speed of removing the mould, the time at which the measurement of the test outcome was taken, the surface on which the mini-slump testing was performed, and the aspect ratio of the mini-slump test mould.

However, the time taken to measure the outcome of the test does have an influence for the study of cementitious materials. The paste studied in the work of Pashias et al. [28] was red mud, which does not show the same time-dependent rheology as do cementitious materials. Cementitious pastes undergo chemical reactions leading to the build-up of a complex microstructure, particles can also aggregate or flocculate, and these factors may all influence the spread size measured during mini-slump testing. Each mini-slump test in this study was carried out in triplicate on individually mixed batches to obtain an average value of the spread size, rather than the common practice in the literature of measuring three data points in succession from a single batch of paste. This removes the possibility of introducing errors through the time dependence (known as ‘slump loss’ in concrete practice) of the yield stress of the reacting paste.

**Fig. 9:**
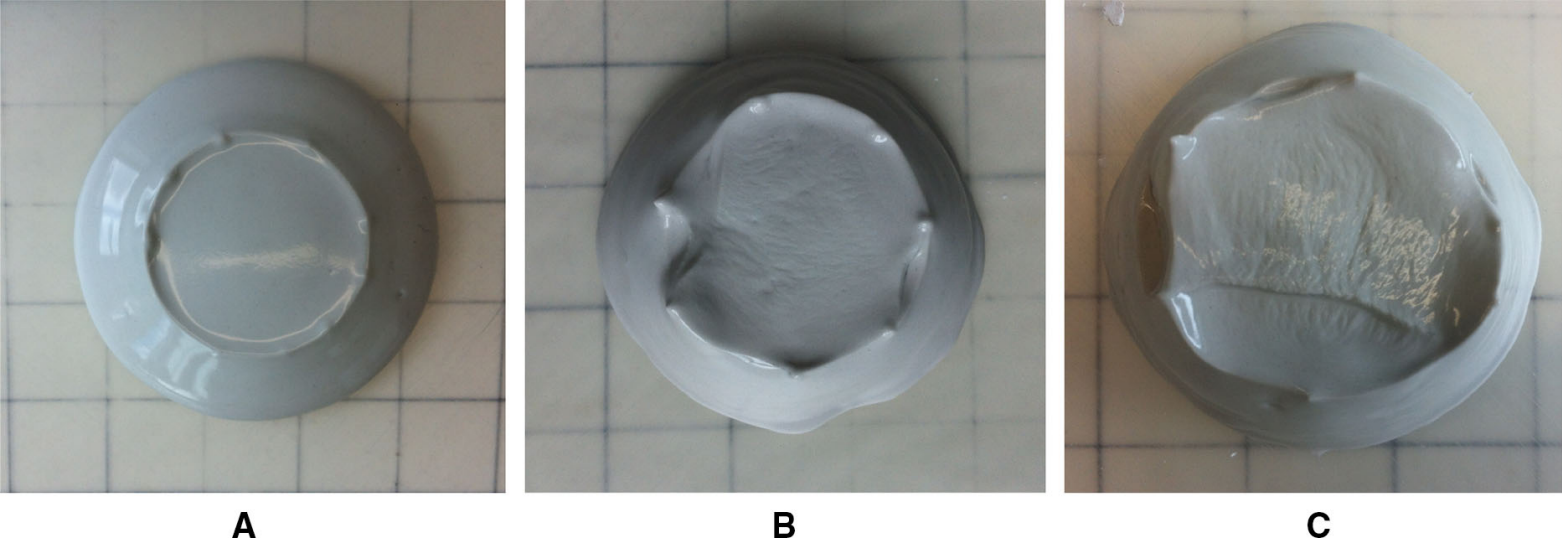
The spread shapes of a paste with 100% slag, *w*/*b* = 0.40, and 4% activator dose, at different times, **a** tested immediately after mixing, **b** 5 min after mixing, **c** 10 min after mixing

To illustrate the importance of the use of separate paste batches in obtaining reproducible measurements in the current study, Fig. 9 presents the spread shapes of the same mixed paste (100% slag, *w*/*b* = 0.40 and 4% activator dose) measured immediately after mixing, and with 5 and 10 min delays before measurement. The dramatic change in the pat shape during this short timeframe demonstrates the importance of the structural evolution of the paste even in the first minutes after mixing, which may also include some loss of water from the surface due to drying effects. The images in Fig. 9 highlight the importance of accurate timing of the mini-slump test. This is why it is proposed in this study to mix the paste separately three times to measure three spread sizes for the same paste, to produce results with optimal reproducibility.

It is also worthwhile to note that the rapid removal of the mould from the paste may introduce additional inertial effects to the final spread. The stress which can be caused by inertial effects is in the range of several Pa, which becomes significant in the case of pastes with very low yield stresses [29], e.g. < 10 Pa. Rapid removal of the mould may also disturb the nature and shape of the paste when performing mini-slump testing, particularly if there is some degree of adhesion between the paste and the mould material, although this was minimised in the current work through the use of a poly(tetrafluoroethene) mould. For the sake of practical experimental operation, the speed of removal of the mould should be slow enough to minimise the inertial effects.

In this study, the aspect ratio of the mini-slump test cone is 1.5, which is in accordance with the Abrams cone geometry widely used in the field of construction materials, but is higher than the aspect ratio recommended by Pashias et al. [28], which was approximately 1.0. Those authors explained that an aspect value too much larger than 1.0 may lead to the collapse of a cylindrical paste specimen, rather than the material flowing. However, no collapse of the pastes was observed here, which means that the geometry of the cone was satisfactory for all of the pastes in this study.

### The influence of mixing protocol on the results of mini-slump testing

**Fig. 10:**
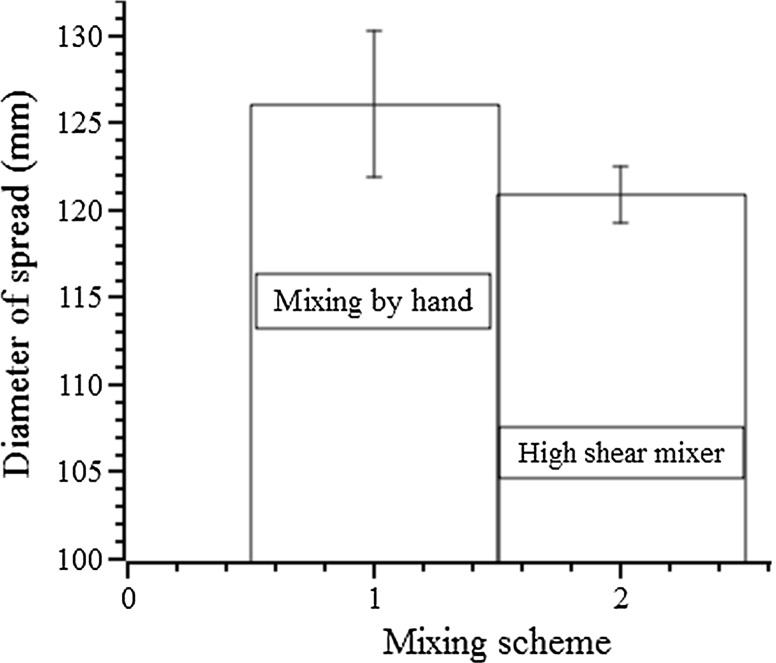
Spread diameter of a paste with 100% slag, *w*/*b* = 0.40, and activator dose 12%, mixed by hand (~ 150 rpm for 2 min) and using the high shear mixer (400 rpm for 2 min)

To investigate the influence of the mixing protocol on the results of mini-slump testing, different possible mixing schemes were studied. The first comparison is the difference between hand mixing and high shear mixing applied to the paste. Figure 10 shows the mini-slump results for a paste with 100% slag, *w*/*b* = 0.40, and activator dose 12%, mixed by hand (2 min) and using a high shear mixer (400 rpm for 2 min). The spread diameter after hand mixing is 126.1 ± 4.2 mm, while high shear mixing gave 120.9 ± 1.6 mm, showing that both the spread value and reproducibility of the tests were influenced by the choice of mixing method.

**Fig. 11:**
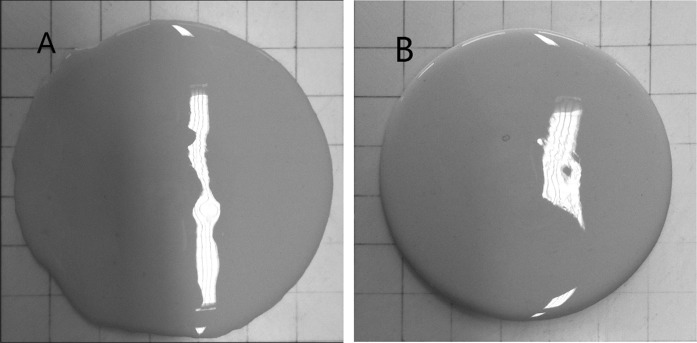
The outline of the paste (100% slag, *w*/*b* 0.40 and activator dose 12%) mixed by hand (**a**) and using the high shear mixer (**b**) after mini-slump testing

These experimental results demonstrate that mixing by hand was not sufficient to disperse the precursor particles in the aqueous environment (water + activator solution) used here. The speed of (vigorous) hand mixing in this study was approximately 150 rpm, which is much lower than the 400 rpm generated by the high shear mixer. Apart from the lower speed, the paddle used in hand mixing was also much less efficient than the dedicated shear blade of the high shear mixer. The larger spread diameter resulting from hand mixing could be attributed to the insufficient mixing that is not strong enough to evenly disperse particles in the paste, allowing the liquid activator and water to flow unevenly (i.e. reach a larger spread diameter in the longest dimension) when performing mini-slump testing. The pictures in Fig. 11 depict the shapes of the two identical pastes (100% slag, *w*/*b* 0.40 and activator dose 12%) after mini-slump testing, where the pastes were mixed by hand and by the high shear mixer, respectively. The shape obtained following hand mixing appeared obviously less circular than the one mixed at high shear, demonstrating the particles in the paste mixed by hand was not evenly dispersed due to the insufficient mixing intensity. This loss of pat circularity for hand-mixed pastes was observed consistently across multiple tests.

Experimental work by Han and Ferron [30] demonstrated that all mixtures containing superplasticisers prepared using the ASTM C1738 protocol for high-shear mixing [31] displayed higher yield stresses than their counterpart pastes prepared according to the normal-shear ASTM C305 protocol [32]. The results obtained here agree well with these previous observations, in that a smaller mini-slump spread size was observed for the paste mixed by high shear mixing in this study, and higher yield stress corresponds to smaller spread size [30, 33]. However, Roy and Asaga [34] reported that increasing intensity of mixing caused a breakdown of particulate aggregates and thus substantially decreased yield stress; the results obtained here were not consistent with that interpretation.

The coefficient of variation of spread size of this paste mixed by hand (3.3%) is also much higher than that achieved with the high shear mixer (1.3%), indicating that high shear mixing produces more reproducible mini-slump test results than hand mixing, as the energy and mode of mixing energy input are better controlled using a mechanical mixer.

Table 4 shows the spread size of a paste with 75% slag + 25% fly ash, *w*/*b* = 0.40 and activator dose 8%, mixed by a high shear mixer at different speeds, for different durations and with different batch sizes. The combination of these factors generated different mixing energy densities.

**Table 4:** The spread diameter (mm) of pastes with 75% slag + 25% fly ash, *w*/*b* = 0.40 and activator dose 8%, produced at different mixing speeds, durations and batch sizes

400 rpm, 60 g precursor, 2 min	400 rpm, 270 g precursor, 2 min	800 rpm, 270 g precursor, 4 min
109.4 (set 1); 110.2 (set 2)	109.0	110.3

The first (109.4 mm) value in Table 4 was measured for a paste mixed by the mixing protocol which is adopted for all 27 samples in this study, i.e. 60 g precursor, 400 rpm for 2 min. It is interesting to note that in re-testing using the same materials and mixing scheme 12 months after the initial tests (denoted ‘set 2’, where the initial test is ‘set 1’), a very similar value (110.2 mm) was measured. This agreement shows that the single-operator reproducibility of the measurement procedure is high.

When the paste was mixed in a 270 g batch at 400 rpm for 2 min, a spread diameter of 109.0 mm was obtained. Since the mixing speed and time duration were as same as the baseline mixing scheme, each unit of paste in this larger batch received approximately 22% of the mixing energy of the 60 g batch size. However, the spread sizes of the two pastes are comparable, which means that a mixing speed of 400 rpm is also adequate to disperse particles in the paste with this formulation in the larger batch size. Doubling both the speed and mixing duration (final entry in Table 4) for the 270 g batch size again gave a mini-slump result matching the results obtained at lower mixing energy density to within 1%. These results indicate that once a satisfactory mixing condition is determined for a particular paste (i.e. resulting in a well-mixed condition), the test method presented here is satisfactorily robust to variability in mixing parameters to enable it to be a useful and reliable protocol in practice.

## Conclusions

The study investigated the measurement of yield stress of cementitious pastes based on a reproducible mini-slump testing method using a well-defined mixing procedure. The relationship between the spread diameter in mini-slump testing and yield stress was verified with results obtained using a rotational viscometer; the correlation between the two methods is good for low yield stress values, but shows deviations at higher yield stresses when the flow patterns and resultant pat shape in the mini-slump test no longer correspond to the assumptions inherent in the derivation of the governing equation. The work in this paper thus contributes particularly to enabling inexpensive and rapid measurements of the rheology of cementitious pastes with low yield stresses which are difficult to directly evaluate using conventional viscometers. The main conclusions drawn are:

1. The setup and procedure proposed in this study for mini-slump test, i.e. mixing protocol, volume of paste, and the use of a fresh batch of sample for every replicate test, give highly reproducible spread diameters for pastes, which can then be used to calculate yield stress values.
2. The yield stresses calculated based on the spread size from mini-slump test correlate well with the results from conventional viscometry when the paste shows pure shear flow.
3. A controllable high shear mixer performs better than hand mixing, reducing the scatter of mini-slump test results, while the results are relatively robust to variations in batch size, mixing speed and duration.

The results obtained here were based on the analysis of a set of alkali-activated pastes which were designed to span a wide range of yield stress values, from 1.0 Pa to more than 50 Pa; the best results were obtained below 10 Pa which is a regime of significant interest in the study of both traditional and non-traditional cementitious paste systems.
